# LRP1B associated with immune cell infiltration influenced the efficacy of immunotherapy in colorectal cancer patients

**DOI:** 10.1016/j.clinsp.2024.100516

**Published:** 2024-11-11

**Authors:** Weiming Weng, Shengquan He, Guoxiong Zhang, Xindong Zhou, Kang Li, Jiajun Lai

**Affiliations:** Department of Gastrointestinal Surgery, The Yue Bei People's Hospital of Shaoguan, No. 133, Huimin South Road, Shaoguan, Guangdong 512026, China

**Keywords:** Colorectal cancer, Next-generation sequencing, Gene mutation, LRP1B, Immune infiltration

## Abstract

**Objective:**

Colorectal cancer is one of the most common malignant tumors in the world, which seriously threatens human health. It is essential for the search for new oncogene targets in colorectal cancer.

**Methods:**

Samples from 57 colorectal cancer patients were collected in this study. Next-Generation Sequencing (NGS) was performed to detect gene mutation, assess Microsatellite Instability (MSI), and evaluate Tumor Mutational Burden (TMB). RNA data from 528 CRC patients from the TCGA database were analyzed.

**Results:**

A total of 57 colon cancer patients were included in this study, including 30 males and 27 females, with a mean age of 56 years. In this study, the most common mutations were APC (79 %), TP53 (61 %), TTN (48 %), KRAS (42 %), SYNE1 (28 %), MUC16 (25 %), PIK3CA (25 %), FAT4 (22 %), RYR2 (19 %), OBSCN (18 %), and ZFHX4 (18 %). Subsequently, the authors analyzed gene mutations in colorectal cancer patients according to gender, age, and TMB status. Significant differences in immune cell infiltration were found between colorectal cancer tissues and normal tissues by CIBERSORT analysis. LRP1B may serve as a potential colorectal cancer therapeutic target, and its absence leads to changes in immune cell infiltration.

**Conclusion:**

The authors described the molecular characteristics of CRC. Loss of LRP1B leads to changes in immune cell infiltration and can be used as a therapeutic target for colorectal cancer.

## Highlights

• Mutations in the LRP1B gene can affect immune cell infiltration in cancer patients, thereby affecting the effect of clinical immunotherapy.
• It can provide a reference for the emergence of new therapeutic targets for colorectal cancer in clinical practice.
• Loss of LRP1B leads to changes in immune cell infiltration and can be used as a therapeutic target for colorectal cancer.

## Introduction

Malignant tumors are one of the diseases that seriously endanger human health nowadays, and the most common ones are Colorectal Cancer (CRC), adenocarcinoma, liver cancer, and lung cancer.^1^^,^^2^ Colorectal cancer is a highly prevalent malignant tumor in humans, and the incidence and mortality have increased cumulatively every year.^3^^,^^4^ Early detection, early diagnosis, and early treatment of colorectal cancer are particularly important. Early diagnosis methods of colorectal cancer generally include ultrasound, radiology, isotope, cytology, and serum tumor markers.^5^^,^^6^ In recent years, the therapeutic efficacy of colorectal cancer has been increasing, and the treatment methods include traditional surgery, chemoradiotherapy, and emerging cancer immunotherapy.^7^ The occurrence, development, metastasis, recurrence, and prognosis of colorectal cancer are not determined by a single factor, but regulated by a variety of factors and multiple genes.^8^ In recent years, cancer research has developed rapidly, and a series of major discoveries such as oncogenes, tumor suppressor genes, signal transduction mechanisms, apoptosis, and tumor angiogenesis have developed cancer research to the molecular biology level.^9^^,^^10^

In terms of the pathogenesis of colorectal cancer, more than 90 % of colorectal cancers develop from adenoma-carcinoma, and genetic and molecular changes lead to the transformation of normal intestinal epithelial cells into adenoma cells, which eventually lead to the development of adenoma cells into carcinoma when abnormal molecules gradually accumulate.^11^ During this process, a large number of genetic mutations emerge. At the same time, with the gradual deepening of precision medicine, more and more studies have found the relationship between colorectal cancer and gene mutations, and the research and development of many novel drugs have also focused on targeted drugs for specific genes. For example, panitumumab, cetuximab, and other drugs can be used in patients with wild-type KRAS/NRAS/BRAFV600E activating mutation in the left colon cancer.^12^^,^^13^ Platinum drugs (cisplatin, carboplatin, oxaliplatin, etc.) may have better efficacy in patients with mutations in ERCC1, GSTT1, XRCC1, and other genes than wild-type.^14^ Nivolumab alone or nivolumab combined with ipilimumab can be used in patients with MSI-H or dMMR metastatic colorectal cancer after the progression of standard chemotherapy.^15^ The detection of variant genes is a prerequisite for targeted therapy in colorectal cancer patients. In recent years, molecular marker detection technology has developed rapidly in the medical field. Because of its comprehensive advantages in tumor gene mutation detection, next-generation sequencing technology has shown more and more broad application prospects in the field of colorectal cancer treatment because of its high throughput, low cost, and high efficiency.^16^

In this study, Next-Generation Sequencing (NGS) was used to detect the mutated genes in clinical colorectal cancer patients and map the mutated genes in colorectal cancer patients, which can provide a reference for targeted therapy in colorectal cancer patients.

## Materials and methods

### Study subjects

In this study, the authors collected 57 samples from patients with colorectal cancer who visited YueBei People's Hospital from August 2019 to May 2023. RNA data from 528 CRC patients from the TCGA database were analyzed. The study was approved by the hospital ethics committee. Informed consent was obtained from all patients. For Formalin-Fixed and Paraffin-Embedded (FFPE) samples, tissues were processed and stained with Hematoxylin and Eosin (HampE) to confirm the presence of tumor cells (70 % or greater).

### Tissue DNA extraction

DNA was extracted from tumor tissues using a QIAamp DNA FFPE tissue kit (Qiagen; CA, USA). The nucleic acid concentration was determined using a NanoDrop1000 spectrophotometer (Thermo Fisher Scientific; Waltham, MA, USA).

### NGS sequencing

Covaris M220 focused ultrasound is used for fragmentation of extracted DNA and requires a minimum of 50 ng of DNA. End repair, phosphorylation, and adapter ligation were subsequently performed. Screening for DNA fragments of 200‒400 bp was performed using the Agencourt AMPure XP Kit. Polymerase Chain Reaction (PCR) amplification was subsequently performed. DNA fragments 200–400 bp in size were selected using magnetic beads (Agencourt AMPure XP Kit), then hybridized to capture probe baits, hybridized to magnetic beads, and amplified by Polymerase Chain Reaction (PCR). Fragments were assessed for quality and size using a Qubit 2.0 fluorometer and dsDNA High Sensitivity Assay Kit (Life Technologies, Carlsbad, CA). The index samples were sequenced with paired-end reads on a NextSeq 500 sequencing system (Illumina, Inc., USA). NGS sequencing was performed by Zhuhai Sanmed Gene Diagnostics Ltd.

### Statistical analysis

Data analyses were performed using R (version 4.0.5, 2021) and the GraphPad Prism software (version 7.01). Data were presented as the mean ± Standard (SD). Differences between the two groups were analyzed using the unpaired Student *t*-test Statistical significance was set to a *p <* 0.05.

## Results

### Basic information

A total of 57 patients (30 males and 27 females, mean age: 56±11 years) with colon cancer were enrolled in this study. 20 patients were TMB-H and 37 patients were TMB-L. Left-sided colorectal cancer patients were 36 and Right-sided colorectal cancer patients were 21. MSS patients were 54 and MSI-H patients were 3 (Table 1).

**Table 1:** Basic information of patients.

Item	Number of cases ( %)
Gender	
Male	901 (70.23 %)
Female	383 (29.77 %)
Age (year)	56+11
TMB	
TMB-H	961 (74.90 %)
TMB-L	323 (25.10 %)
Histology	
Left-sided colorectal	582 (76.48 %)
Right-sided colorectal	179 (23.52 %)
MSI status	
MSS	1076 (82.90 %)
MSI-H	222 (17.10 %)
MSI-L	0

### Gene mutations of CRC

**Fig. 1:**
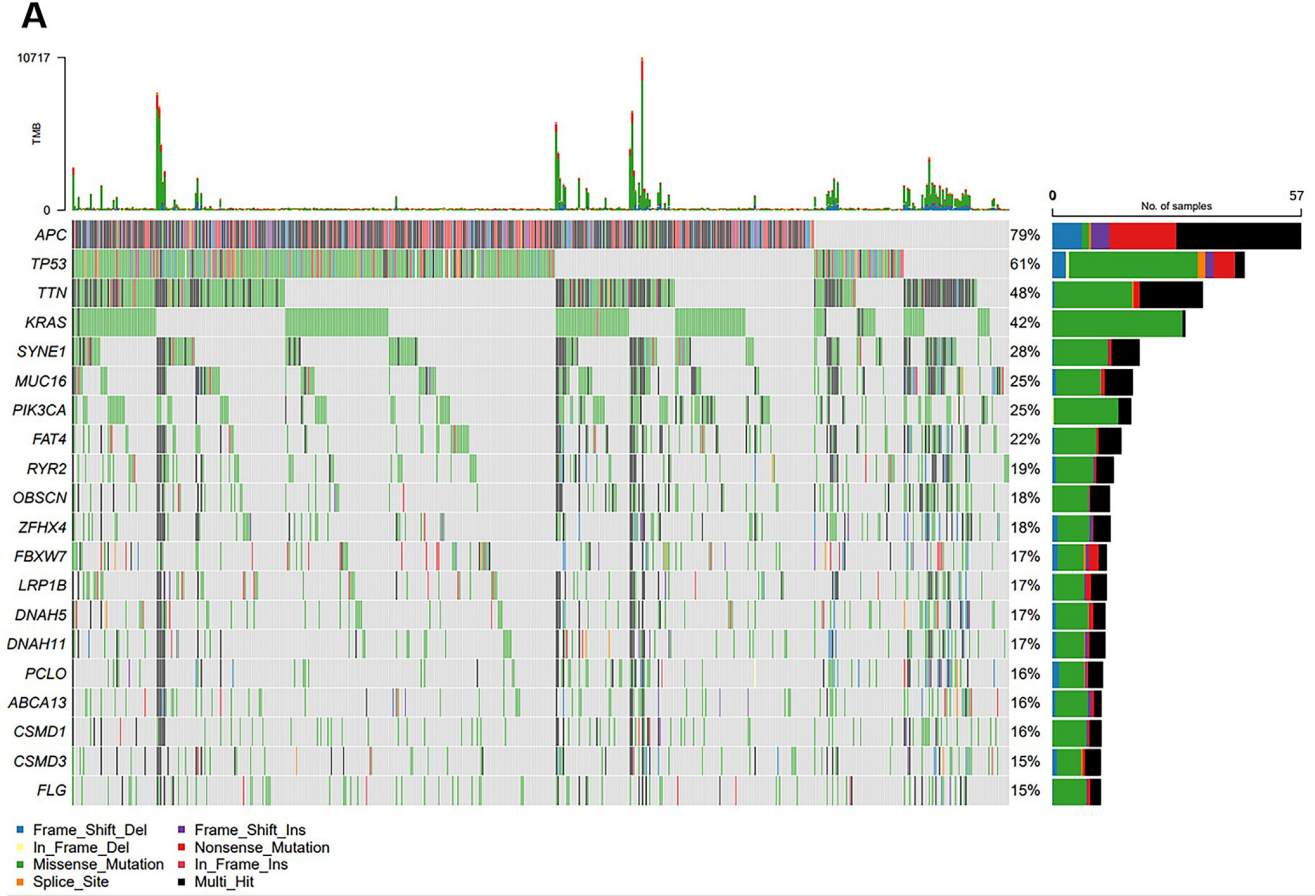
**Gene mutations of CRC.** Mutational landscape of CRC patients.

In this study, the most common mutations were APC (79 %), TP53 (61 %), TTN (48 %), KRAS (42 %), SYNE1 (28 %), MUC16 (25 %), PIK3CA (25 %), FAT4 (22 %), RYR2 (19 %), OBSCN (18 %), and ZFHX4 (18 %) (Fig. 1).

### Gene mutations of CRC in different genders

**Fig. 2:**
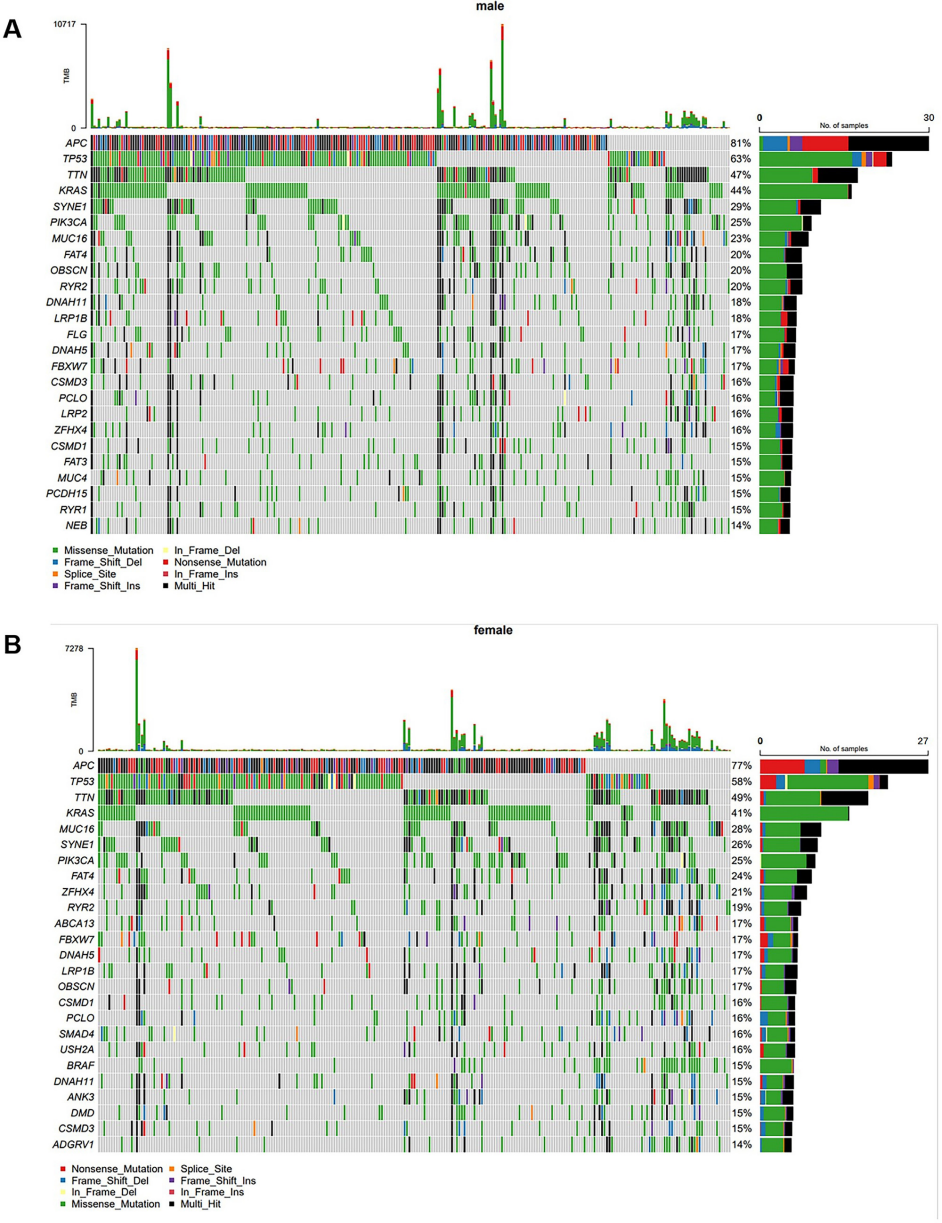
**Gene mutations of CRC in different genders.** (A) Mutational landscape of male CRC patients. (B) Mutational landscape of female CRC patients.

Subsequently, the authors performed a Gas analysis on CRC patients of different genders. The results showed that the most common mutations were APC (81 %), TP53 (63 %), TTN (47 %), KRAS (44 %), SYNE1 (29 %), PIK3CA (25 %), MUC16 (23 %), FAT4 (20 %), OBSCN (20 %), and RYR2 (20 %) in male CRC patients (Fig. 2A). In female CRC patients, the most common mutations were APC (77 %), TP53 (58 %), TTN (49 %), KRAS (41 %), MUC16 (28 %), SYNE1 (26 %), PIK3CA (25 %), FAT4 (24 % %), ZFHX4 (21 %), and RYR2 (19 %) (Fig. 2B).

### Gene mutations of CRC in different ages

**Fig. 3:**
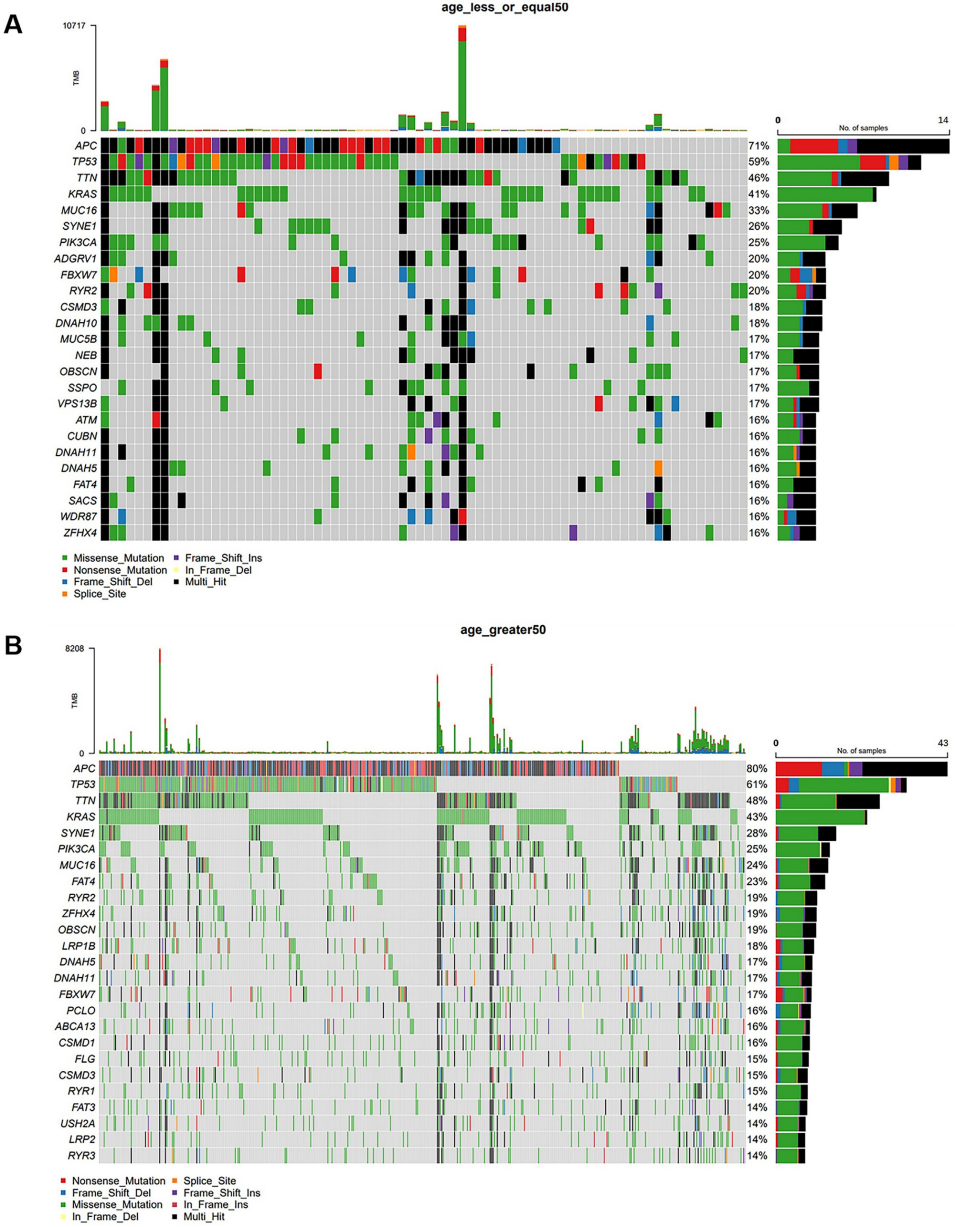
**Gene mutations of CRC in different ages.** (A) Mutational landscape of CRC patients who > 50-years. (B) Mutational landscape of female CRC patients who ≤ 50-years.

The authors divided CRC patients into groups older than 50 years and those younger than or equal to 50 years using the age limit of 50 years. In the group of CRC patients older than 50 years, the most common mutations were APC (71 %), TP53 (59 %), TTN (46 %), KRAS (41 %), MUC16 (33 %), SYNE1 (26 %), PIK3CA (25 %), ADGRV1 (20 %), FBXW7 (20 %), and RYR2 (20 %) (Fig. 3A). In the group of CRC patients aged 50 years or younger, the most common mutations were APC (80 %), TP53 (61 %), TTN (48 %), KRAS (43 %), SYNE1 (28 %), PIK3CA (25 %), MUC16 (24 %), FAT4 (23 %), RYR2 (19 %), ZFHX4 (19 %), and OBSCN (19 %) (Fig. 3B).

### GAs of CRC in TMB-H and TMB-L patients

**Fig. 4:**
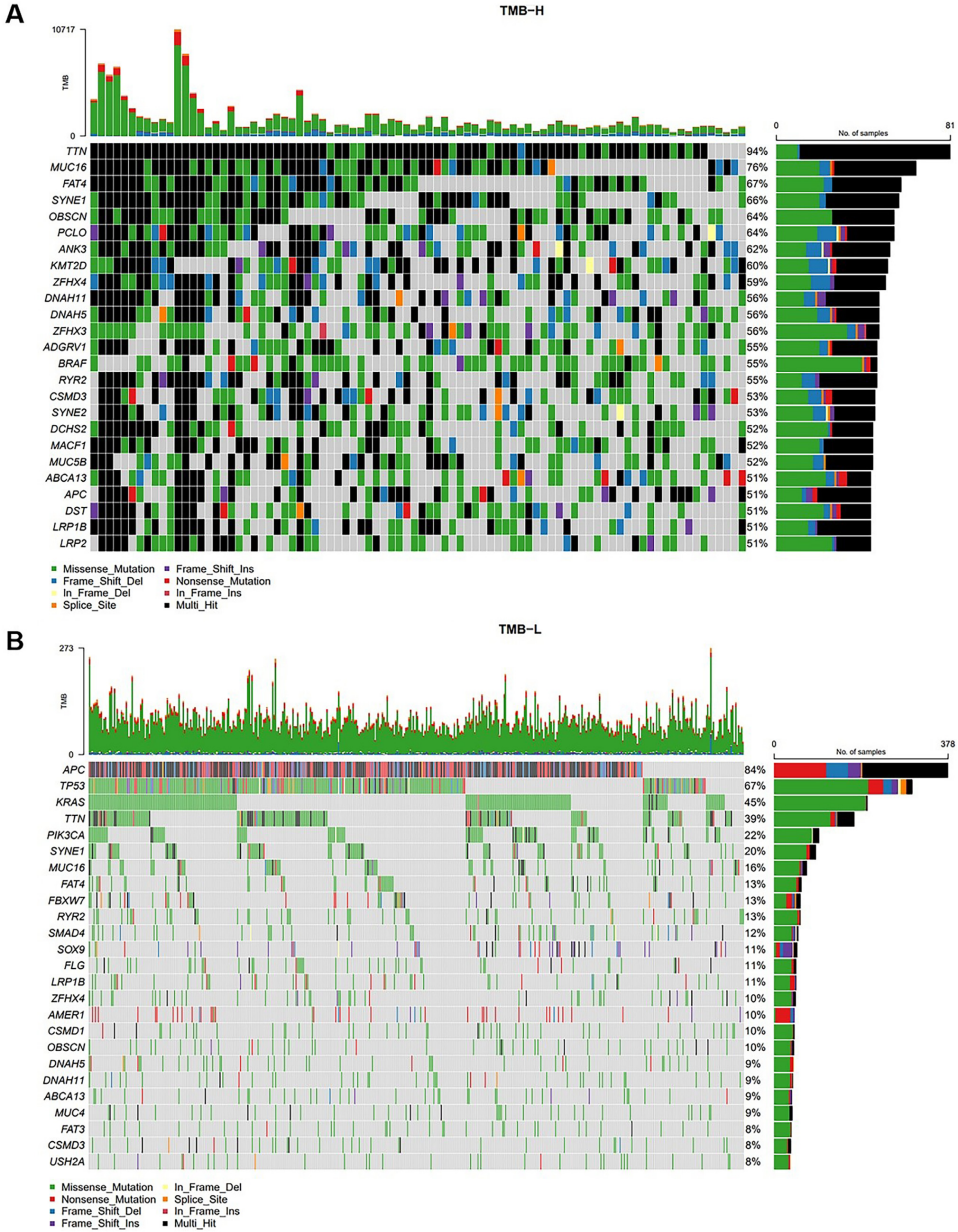
**Gene mutations of CRC in TMB-H and TMB-L.** (A) Mutational landscape of CRC patients who were TMB-H. (B) Mutational landscape of female CRC patients who were TMB-L.

The authors subsequently divided CRC patients into TMB-H and TMB-L groups according to TMB status. In the TMB-H group, the most common mutations were: TTN (94 %), MUC16 (76 %), FAT4 (67 %), SYNE1 (66 %), OBSCN (64 %), PCLO (64 %), ANK3 (62 %), KMT2D (60 %), ZFHX4 (59 %), DNAF11 (56 %), DNAH5 (56 %), and ZFHX3 (56 %) (Fig. 4A). In the TMB-L group, the most common mutations were APC (84 %), TP53 (67 %), KRAS (45 %), TTN (39 %), PIK3CA (22 %), SYNE1 (20 %), MUC16 (16 %), FAT4 (13 %), FBXW7 (13 %), and RYR2 (13 %) (Fig. 4B).

### LRP1B can affect immune cell infiltration

**Fig. 5:**
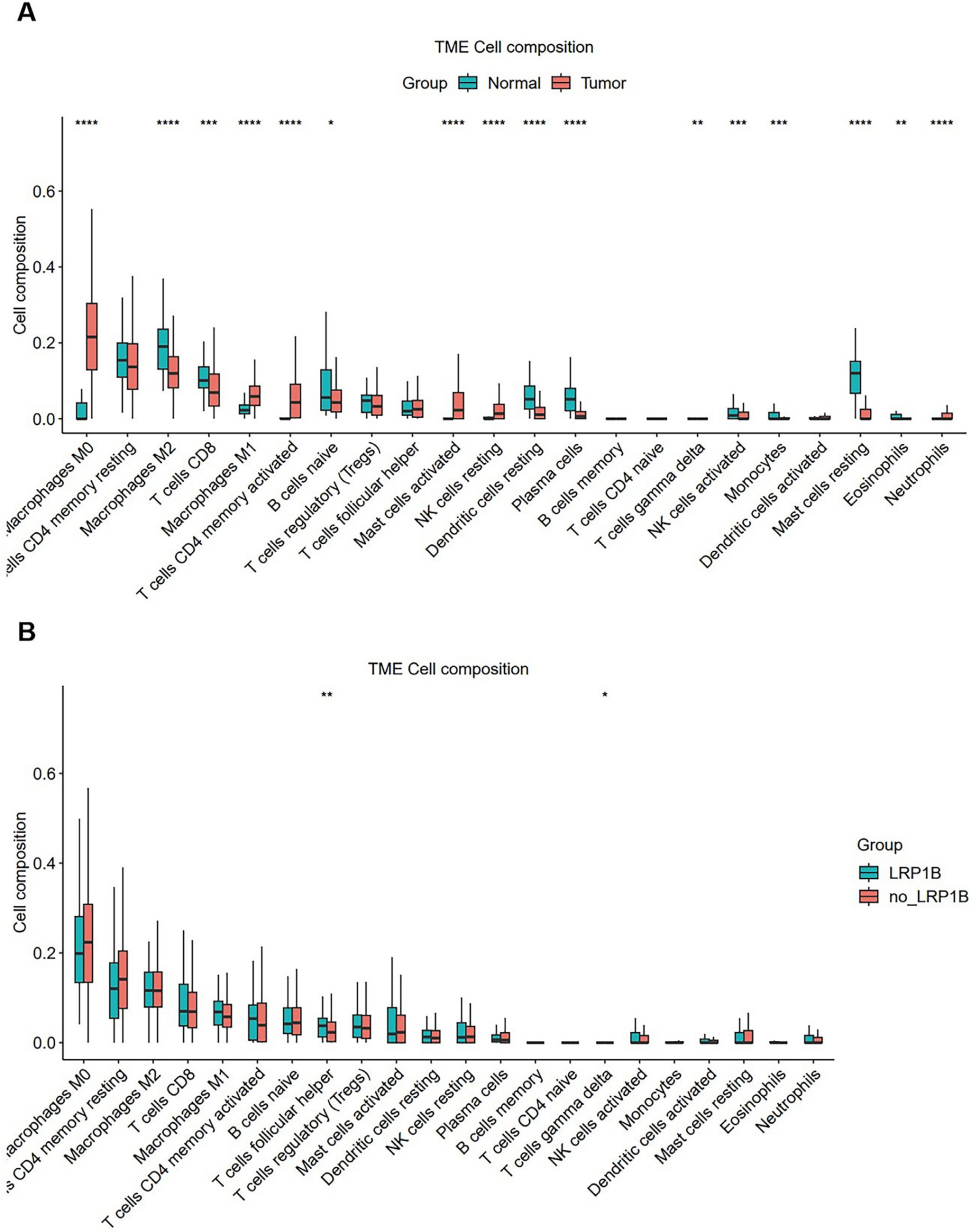
**CIBERSORT analysis.** (A) CIBERSORT analysis of colon cancer tissues versus normal tissues. (B) CIBERSORT analysis of LRP1B normal tissues versus LRP1B null tissues (* *p <* 0.05; ** *p <* 0.01; *** *p <* 0.001; **** *p <* 0.0001).

Through analysis of the above genetic mutation data, the authors found that LRP1B is a potential therapeutic target for colorectal cancer. In this study, the mutation frequency of LRP1B in colorectal cancer was 17 % (Fig. 1A). TMB-H occurred in 67 % of patients in the LRP1B mutation group and 26 % of patients in wild-type LRP1B (*p =* 0.0179). LRP1B mutations were associated with high TMB (Fig. 4). The incidence of MSI-H was 16.7 % in the LRP1B mutation group and 0 in wild-type LRP1B patients (*p =* 0.04286). LRP1B mutation was more likely to occur in MSI-H patients. The incidence of PD-L1 > 1 % was 80 % in the LRP1B mutation group and 26.7 % in the LRP1B wild-type group (*p =* 0.0068). LRP1B mutations were more likely to occur in PD-L1-positive patients. The above results indicated that LRP1B can be used as one of the factors to predict the efficacy of immunotherapy in CRC. CIBERSORT analysis from TCGA database revealed a significant difference in immune cell infiltration between colon cancer tissues and normal tissues (Fig. 5A). LRP1B may serve as a potential colon cancer therapeutic target, and its absence leads to changes in immune cell infiltration (Fig. 5B).

## Discussion

Colorectal cancer is a malignant intestinal lesion that occurs in the colorectal mucosal epithelium under the combined action of environmental and or genetic factors. The latest global cancer statistics show that colorectal cancer is the third most common cancer in the world, with the mortality rate ranking second in the world. In 2020, there were about 1.9 million new cases and 935,000 deaths worldwide.^17^ In China, the latest survey data also shows that there are 560,000 new cases of colorectal cancer and about 290,000 deaths, which is the most common malignant tumor of the digestive tract in China.^18^

After cells sense the stimulation of information molecules through cell membranes or intracellular receptors, they switch through intracellular signal transduction systems, and the process leading to cell biological function is called cell signal transduction.^19^ Signaling pathways regulate the growth, proliferation, differentiation, apoptosis, and other activities of body cells, and abnormal activation of signaling pathways or changes in structural activity are associated with many malignant tumors.^20^ The main signaling pathways in tumors mainly include the JAK-STAT signaling pathway, p53 signaling pathway, canonical Wnt signaling pathway, Ras signaling pathway, BMP signaling pathway, Notch signaling pathway, etc.^21^, ^22^, ^23^ Through a series of studies, it has been found that these signaling pathways play a crucial role in a variety of malignant tumors. Genetic alterations in the WNT, RAS-MAPK, PI3K, TGF-β, and p53 pathways are common in CRC.^24^^,^^25^ The widespread use of NGS in the clinic has made it possible to identify genetic mutations in colorectal cancer on a large scale. Several articles have now reported on the use of NGS for the identification of colorectal cancer gene mutations.^26^, ^27^, ^28^

Liu et al.^29^ identified the most frequently mutated genes of Chinese CRC patients to be TP53, APC, KRAS, SMAD4, FBXW7, and PIK3CA.In this study, however, the most common mutations were APC (79 %), TP53 (61 %), TTN (48 %), KRAS (42 %), SYNE1 (28 %), MUC16 (25 %), PIK3CA (25 %), FAT4 (22 %), RYR2 (19 %), OBSCN (18 %), and ZFHX4 (18 %). This may be related to the number of patients included in this study, which included a total sample of 57 colon cancer patients. Similarly, Huang et al.^30^ performed NGS sequencing using samples from 630 Chinese colorectal cancer patients and showed different molecular profiles in Right-sided and Left-sided Colorectal Cancer (RCC and LCC) patients. Hu et al.^31^ used NGS sequencing to compare genetic mutations in colorectal cancer patients who develop liver or lung metastases and provided a molecular basis for the study of colorectal cancer metastasis.

Lipoprotein Receptor-Related Protein 1B (LRP1B) is a newly discovered candidate tumor suppressor gene. Current studies have shown that LRP1B is widely expressed in normal human tissues and tumor cells, and can regulate tumor cell proliferation, adhesion, differentiation, and angiogenesis.^32^^,^^33^ It has been reported that LRP1B was prevalent in a variety of malignancies, such as colon cancer, breast cancer, and medulloblastoma.^34^, ^35^, ^36^ Zhuang et al.^37^ reported that LRP1B could be used as one of the prognostic factors in colorectal cancer. Wang et al.^34^ reported that the knockdown of LRP1B in colon cancer cell lines inhibited the growth and migration of colon cancer cells by affecting the binding of DVL2 to AXIN. However, there are still few studies on the clinical relevance of LRP1B in different populations. In this study, the authors analyzed NGS sequencing using clinical patient samples combined with TCGA data and found that LRP1B was associated with immune infiltration in Chinese clinical CRC patients. It has been reported that LRP1B gene mutation was associated with immune infiltration in a variety of tumors.^38^, ^39^, ^40^ However, there were no reports between LRP1B gene mutation and LRP1B gene mutation in CRC. Therefore, the relationship between LRP1B gene mutation and immune infiltration of CRC tumors in the Chinese CRC patient population remains to be further investigated.

However, some limitations remain in this study. Firstly, this study found that LRP1B could affect immune cell infiltration, but this was not confirmed experimentally, and more experiments were required to subsequently confirm the effect of LRP1B. Secondly, this study lacks data on pathological grades due to missing partial information.

## Conclusion

In this study, 57 colorectal cancer patient samples were used to explore the mutated genes of colorectal cancer and map the gene mutations in colorectal cancer patients. Mutations in the LRP1B gene can affect immune cell infiltration in cancer patients, thereby affecting the effect of clinical immunotherapy. It can provide a reference for the emergence of new therapeutic targets for colorectal cancer in clinical practice.

## Declarations

### Ethics approval and consent to participate

This study was conducted in accordance with the declaration of Helsinki. This study was conducted with approval from the Ethics Committee of The YueBei People's Hospital of Shaoguan (KY-2021-160). Written informed consent was obtained from all participants.

### Consent to participate

Written informed consent was obtained from all participants.

### Availability of data and materials

All data generated or analyzed during this study are included in this published article.

### Consent for publication

The manuscript is not submitted for publication or consideration elsewhere.

## Funding

This research did not receive any funding support.

## CRediT authorship contribution statement

**Weiming Weng:** Conceptualization, Visualization, Data curation, Writing – original draft, Writing – review & editing. **Shengquan He:** Conceptualization, Visualization, Formal analysis, Writing – original draft, Writing – review & editing. **Guoxiong Zhang:** Project administration, Formal analysis, Writing – original draft, Writing – review & editing. **Xindong Zhou:** Project administration, Methodology, Data curation, Writing – original draft, Writing – review & editing. **Kang Li:** Project administration, Methodology, Data curation, Writing – original draft, Writing – review & editing. **Jiajun Lai:** Methodology, Formal analysis, Writing – original draft, Writing – review & editing.

## Declaration of competing interest

The authors declare no conflicts of interest.
